# Characterization of ERK Docking Domain Inhibitors that Induce Apoptosis by Targeting Rsk-1 and Caspase-9

**DOI:** 10.1186/1471-2407-11-7

**Published:** 2011-01-10

**Authors:** Sarice R Boston, Rahul Deshmukh, Scott Strome, U Deva Priyakumar, Alexander D MacKerell, Paul Shapiro

**Affiliations:** 1 Department of Pharmaceutical Sciences, School of Pharmacy, University of Maryland 20 N. Pine St. Baltimore, MD 21201 USA; 2 Department of Otorhinolaryngology, School of Medicine, University of Maryland 16 S. Eutaw St. Baltimore, MD 21201 USA

## Abstract

**Background:**

The extracellular signal-regulated kinase-1 and 2 (ERK1/2) proteins play an important role in cancer cell proliferation and survival. ERK1/2 proteins also are important for normal cell functions. Thus, anti-cancer therapies that block all ERK1/2 signaling may result in undesirable toxicity to normal cells. As an alternative, we have used computational and biological approaches to identify low-molecular weight compounds that have the potential to interact with unique ERK1/2 docking sites and selectively inhibit interactions with substrates involved in promoting cell proliferation.

**Methods:**

Colony formation and water soluble tetrazolium salt (WST) assays were used to determine the effects of test compounds on cell proliferation. Changes in phosphorylation and protein expression in response to test compound treatment were examined by immunoblotting and *in vitro *kinase assays. Apoptosis was determined with immunoblotting and caspase activity assays.

**Results:**

*In silico *modeling was used to identify compounds that were structurally similar to a previously identified parent compound, called **76**. From this screen, several compounds, termed **76.2**, **76.3**, and **76.4 **sharing a common thiazolidinedione core with an aminoethyl side group, inhibited proliferation and induced apoptosis of HeLa cells. However, the active compounds were less effective in inhibiting proliferation or inducing apoptosis in non-transformed epithelial cells. Induction of HeLa cell apoptosis appeared to be through intrinsic mechanisms involving caspase-9 activation and decreased phosphorylation of the pro-apoptotic Bad protein. Cell-based and *in vitro *kinase assays indicated that compounds **76.3 **and **76.4 **directly inhibited ERK-mediated phosphorylation of caspase-9 and the p90Rsk-1 kinase, which phosphorylates and inhibits Bad, more effectively than the parent compound **76**. Further examination of the test compound's mechanism of action showed little effects on related MAP kinases or other cell survival proteins.

**Conclusion:**

These findings support the identification of a class of ERK-targeted molecules that can induce apoptosis in transformed cells by inhibiting ERK-mediated phosphorylation and inactivation of pro-apoptotic proteins.

## Background

The extracellular signal-regulated kinases-1 and 2 (ERK1/2) proteins are members of the mitogen activated protein (MAP) kinase superfamily that regulate cell proliferation and survival. ERK1/2-mediated cell survival occurs through protection against apoptosis by inactivating pro-apoptotic proteins. For example, ERK proteins promote cell survival by inhibiting caspase-9 [1,2] or Bim (Bcl-2-interacting mediator of cell death) through direct phosphorylation [3]. Indirect inhibition of apoptosis occurs through ERK phosphorylation and activation of p90Rsk-1, which phosphorylates the pro-apoptotic Bad (Bcl-xL/Bcl-2 associated death promoter) protein and causes 14-3-3-mediated sequestering that prevents interactions with the pro-survival protein Bcl-2 [4,5]. Thus, constitutive activation of the ERK1/2 pathway through mutations in upstream receptors, Ras G-proteins, and kinases, such as B-Raf, provides transformed cancer cells with a survival advantage [6-8].

Significant effort has gone into developing molecules that inhibit proteins in the ERK1/2 pathway [9,10]. These drug discovery efforts include monoclonal antibodies and small molecules that inhibit receptor tyrosine kinases, Ras G-proteins, Raf, or MEK proteins [9,11-13]. Although some of these therapies have shown promising clinical results, toxicity to skin, cardiac, and gastrointestinal tissue has been reported [14,15]. The toxicity associated with upstream inhibition of ERK1/2 signaling is likely due to the effects on the ERK pathway in normal tissue and the various ERK1/2 substrates that regulate cellular functions [6,16]. Thus, inhibition of specific ERK functions, such as regulation of pro-apoptotic proteins, may be an alternative approach to alleviating toxic side effects resulting from complete inhibition of ERK signaling by compounds targeting upstream proteins. To test this, we have identified molecules that act independent of the ATP binding site and are predicted to be selective for ERK1/2 substrate docking domains [17,18]. By developing compounds that are substrate selective, our goal is to inhibit ERK functions that are associated with cancer cell survival but preserve ERK functions in normal non-cancerous cells.

ERK1/2 are proline-directed serine/threonine kinases that phosphorylate substrate protein sequences containing, at minimum, a proline in the +1 position (S/TP site). Proline in the -2 position (PXS/TP sequence) may also determine phosphorylation specificity [19]. While this consensus sequence is shared by the other MAP kinases proteins, including p38 MAP kinases, c-Jun N-terminal kinases (JNKs), and ERK5, each MAP kinase retains substrate specificity suggesting that other determinants of kinase-substrate interactions are involved. Currently, two distinct docking domains on substrates have been identified to mediate interactions between protein substrates and MAP kinases [19-22]. The D-domain or DEJL site (docking site for ERK or JNK, LXL), consists of two or more basic residues, a short peptide linker, and a cluster of hydrophobic residues. ERK1/2 substrates containing D-domains include ELK-1, p90Rsk-1, MKP-3, and caspase-9 [1,23,24]. D-domains have been found on substrates for ERK, JNK, and p38 MAP kinases [25,26]. MAP kinase substrates may also contain an F-site or DEF (docking site for ERK, FXF) motif, which contains the consensus FXFP motif. The F-site is 6-20 amino acids C-terminal to the phosphorylation site [19] is also found on ELK-1 as well as substrates like KSR and nucleoporins [27].

Specific residues on MAP kinases form docking domains that determine binding specificity with substrate proteins. ERK1/2 and other MAP kinases contain a common docking (CD) domain, which includes aspartate residues 316 and 319 (labeled for ERK2) that are located on the side opposite of the TXY activation loop [25] and mediates interactions with the substrate D-domains [27,28]. While the CD domain shares common features among MAP kinases, differences in the CD domains and adjacent residues of ERK1/2 and p38 MAP kinases may be responsible for determining the specificity of substrate interactions [29]. The F-site containing substrates are thought to form hydrophobic interactions with ERK1/2 on a F-site recruitment site (FRS), which consists of leucines 198, 232, 235 and tyrosines 231 and 261 residues (labeled for ERK2) located near the TXY activation site [27].

We have previously utilized computer aided drug design (CADD) to identify low-molecular weight (LMW) compounds that are predicted to interact near the ERK2 CD domain and selectively disrupt ERK2 interactions with substrate proteins [17,18]. These studies identified several compounds that inhibited ERK1/2's ability to phosphorylate selected substrate proteins but not affect the overall activity of the ERK enzyme supporting the goal of using small molecules to inhibit productive protein-protein interactions. Moreover, some of the compounds tested did not appear to affect the ability for the related p38 MAP kinase to phosphorylate substrate proteins [18]. In addition to inhibiting ERK substrate phosphorylation, several compounds identified inhibited cell proliferation. In the current studies, we have identified a class of structurally similar compounds that inhibit cell proliferation and cause rapid induction of apoptosis in transformed cancer cell lines. Apoptosis appeared to occur through an intrinsic mechanism involving the prevention of ERK2-mediated inhibition of caspase-9 and p90Rsk-1 phosphorylation of the pro-apoptotic protein Bad. In addition, transformed cells appeared more sensitive to the growth inhibitory effects of the test compounds as compared to non-transformed epithelial cells. These findings support the identification of novel LMW compounds that target ERK1/2 proteins and restore apoptosis responses in cancer cells by preventing phosphorylation events that inactivate pro-apoptotic proteins.

## Methods

### Cells and reagents

HeLa S3 (human cervical carcinoma) and retinal pigment epithelial cells that stably express human telomerase (hTERT-RPE) were purchased from American Type Culture Collection (ATCC, Manassas, VA) and maintained in complete medium consisting of Dulbecco's modified Eagle medium (DMEM) plus 10% fetal bovine serum (FBS, Atlanta Biologicals, Lawrenceville, GA) and antibiotics (Penicillin, 100 U/ml; Streptomycin, 100 μg/ml) (Invitrogen, Carlsbad, CA). Hygromycin B, 0.01 mg/mL, (Roche, Indianapolis, IN) was used to maintain selection for the hTERT-RPE cells. Epidermal growth factor (EGF) and etoposide were purchased from Sigma (St. Louis, MO) and used at final concentrations of 25 ng/ml and 50 μM, respectively. The ERK (pT183/pY185) and α-tubulin antibodies were purchased from Sigma. Antibodies against phosphorylated Bad (pS112 or pS136), total Bad, phosphorylated Rsk-1 (pT573), phosphorylated Akt substrates (RXRXXpS/T), Mcl-1, phosphorylated caspase-9 (pT125), and total caspase-9 were purchased from Cell Signaling (Beverly, MA). Antibodies against poly ADP-ribose polymerase (PARP), total Rsk-1, and ERK5 were purchased from Santa Cruz Biotechnology (Santa Cruz, CA). U0126, SB203580, and Akt Inhibitor I were purchased from Calbiochem and used at final concentrations of 10 μM, 20 μM, and 25 μM, respectively. LY294002 was purchased from Cell Signaling and used at a final concentration of 25 μM. Test compounds were purchased from Chembridge (San Diego, CA) and stored as 25 mM stock solutions in DMSO. The general caspase inhibitor, Z-VAD-FMK, was purchased from BD Biosciences (San Jose, CA) and used at a final concentration of 20 μM.

### Protein expression

Plasmids for mammalian and bacterial expression of caspase-9 (catalytically inactive C287A mutant) were purchased from Addgene (catalog #11819 and 11830). The His_6_-tagged caspase-9 (C287A) was purified from BL21(DE3) cells as described [30]. Briefly, BL21(DE3) cells were induced with 0.2 mM isopropyl β-D-1-thiogalactopyranoside for 4 hours and harvested with BugBuster protein extraction reagent (EMD Biosciences, San Diego, CA). Lysates were loaded onto a Talon Co^2+^- IMAC affinity resin column (BD Biosciences, San Jose, CA) and eluted with imidazole. GST-p90Rsk-1 was expressed and purified as previously described [31]. His_6_-tagged ERK was expressed and purified as previously described [17]. The plasmid for Bad was provided by Dr. Michael Greenberg (Harvard University). Transient expression of Bad and caspase 9 (C287A) in HeLa cells was done using Lipofectamine (Invitrogen).

### Immunoblotting

Cells were washed with cold phosphate buffered saline (PBS, pH 7.2; Invitrogen) and protein lysates were collected with 2× SDS-PAGE sample buffer (4% SDS, 5.7 M β-mercaptoethanol, 0.2 M Tris pH 6.8, 20% glycerol, 5 mM EDTA) or cold tissue lysis buffer (TLB; 20 mM Tris-HCl pH 7.4, 137 mM NaCl, 2 mM EDTA, 1% Triton X-100, 0.1% SDS, 25 mM β-glycerophosphate, 2 mM sodium pyrophosphate, 10% glycerol, 1 mM sodium orthovanadate, 1 mM phenylmethylsulfonyl fluoride, 1 mM benzamidine). Lysates collected in TLB were centrifuged at 20,000 (× g) to remove insoluble material and then diluted with an equal volume of 2× SDS-sample buffer. Proteins were separated by SDS-PAGE and analyzed by immunoblotting using enhanced chemiluminesence (ECL, GE Healthcare, United Kingdom). The relative protein levels were determined by densitometry scanning (Alpha Innotech), keeping the pixel intensity within the linear range of detection.

### Fluorescence Quenching Assay

Fluorescence quenching of 1 μM ERK2 was evaluated by spectra analysis (SpectraMax5, Molecular Devices) utilizing an excitation wavelength of 295 nm and the emission spectra was monitored from 300 to 500 nm in the presence or absence of 25 μM of the indicated test compound. Relative changes in ERK2 fluorescence intensity in presence of test compounds were compared to ERK2 fluorescence in the presence of DMSO vehicle only.

### Cell proliferation assays

Cell proliferation was evaluated by colony formation or by water soluble tetrazolium-1 (WST-1) assays. For colony formation, cells (~250 cells/mL) were plated and allowed to recover for 24 hours before treatment with test compounds. Cells were grown for 10-14 days and colonies that formed (approximately 40 cells or more) were fixed for 10 minutes in 4% paraformaldehyde and stained with 0.2% crystal violet in 20% methanol for 1-2 minutes. The number of colonies in the treated samples was expressed as a percentage of the controls, which consisted of 75, 146, or 154 colonies from 3 separate experiments, respectively. WST-1 assay was done according to manufacturer's instructions and cleavage of WST-1 to formazan by cellular mitochondrial dehydrogenases is used as an indicator of viable cells. Briefly, cells (~250/mL) were seeded in 96 well plates and allowed to recover overnight followed by test compound treatment for 7 days. WST-1 reagent was added and absorbance was read at 450 nm with background readings taken at 650 nm. After background subtraction, values were normalized to the control (DMSO only) cells.

### Kinase assays

Active ERK2 (2 ng, New England BioLabs, Ipswich, MA) was incubated with 0.5 μg His_6_-tagged caspase 9 (C287A) or p90RSK-1 for 60 minutes at 30°C in 50 mM Tris-HCl, 10 mM MgCl_2_, 1 mM EGTA, 2 mM DTT, 0.01% Brij 35 (pH 7.5) containing 20 μM ATP and 2 μCi γ-^32^P-ATP. Reactions were stopped with an equal volume of 2× SDS-PAGE sample buffer and the proteins were resolved by SDS-PAGE. The gels were stained with coomassie blue, dried, and ^32^P incorporation into substrate was determined by phosphoimager analysis.

### Caspase activity assays

HeLa cells were seeded at equal density and treated with indicated compound for 5 hours. Cells were harvested and frozen at -20°C. Fluorometric kits (Calbiochem) were used according to manufacturer's instructions to determine the activity of caspases-8 and 9 in cell lysates. Briefly, cell pellets were resuspended with 100 μL sample buffer and incubated on ice for 10 minutes. Samples were centrifuged at 10,000 × g for 10 minutes at 4°C; 50 μL of clarified lysate was transferred to a black walled 96 well plate and 50 μL of assay buffer was added. Fluorescent caspase substrate was added and a baseline reading was taken utilizing 400 nm/505 nm excitation/emission filters. The plates were incubated at 37°C for two hours and read again utilizing the same filters as baseline. Data shown reflects the mean difference ± SEM of relative fluorescence units (RFU) for three independent experiments.

### Annexin-V/Propidium Iodide Staining

HeLa cells were plated at equal density and treated with 50 μM of indicated test compound. Cells were washed and stained according to manufacturers protocol (Calbiochem). Briefly, ~5×10^5 ^cells were incubated for 15 minutes with AnnexinV-FITC. Cells were centrifuged at 1,000 × g for 5 minutes and resuspended in 0.5 mL of cold 1× binding buffer. Propidium Iodide (PI) was added and samples were immediately analyzed by flow cytometry (FACScan Analyser; Becton Dickinson, Franklin Lakes, NJ)

### Statistical Analysis

Comparisons between control or EGF and treated samples were performed utilizing an unpaired students t-test with equal variance using KaleidaGraph software (Synergy Software, Reading, PA). Comparisons within treated groups were performed utilizing a one-way analysis of variance (ANOVA) followed by a Tukey's post hoc analysis with an α-value of 0.05. Statistical significance was indicated with an asterisk (*) if the *p *value was equal to or less than 0.05 or a number sign (#) if the *p *value was equal to or less than 0.01.

## Results

### ERK docking domain inhibitors inhibit cell proliferation

**Figure 1:**
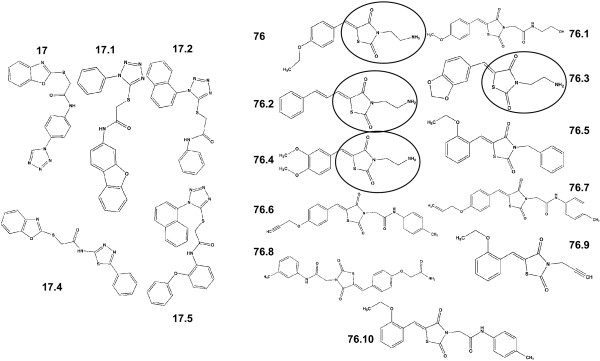
**Structure of test compounds used in these studies**. The area circled represents the thiazolidinedione core with aminoethyl side group that is common to compounds showing similar biological activity

**Figure 2:**
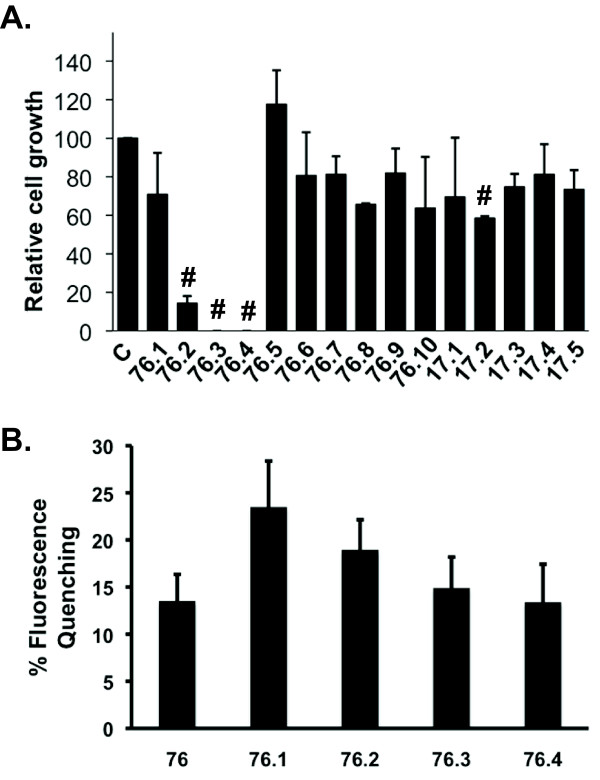
**Effects of ERK targeted docking domain inhibitors on cell proliferation**. (A) HeLa cells were seeded at 250 cells/mL in the presence or absence of 100 uM of the indicated test compound. After 10-14 days, cell colonies were fixed with formaldehyde and stained with crystal violet. Colonies were counted and normalized to the controls for each experiment. Data represent mean ± SD from 3 experiments. # indicates statistical significance compared to untreated controls p ≤ 0.01. C, Control. (B) Fluorescence quenching of ERK2 in the presence of test compounds. Data show the mean ± SD for percent fluorescence quenching as compared to ERK2 fluorescence in the presence of vehicle (DMSO) from 3 independent experiments.

We have previously used computational and cell-based assays to identify LMW compounds that interact with ERK2, inhibit phosphorylation of ERK substrates, and inhibit cell proliferation [17,18,32]. The current studies used a computational search to identify additional compounds that share chemical features with two previously identified active compounds, referred to as **17 **and **76 **[17]. The search was based on the chemical similarity using the MAC-BITS fingerprints in conjunction with the Tanimoto Similarity index to search a virtual database of over 1 million commercially available compounds as described [33]. From this computational search, 5 compounds similar to **17 **and 10 compounds similar to **76 **were selected (Figure 1) and tested for inhibition of cell proliferation. Similar to **76**, test compounds **76.2**, **76.3**, and **76.4 **inhibited proliferation of HeLa cells at 100 μM (Figure 2A) and other transformed cell lines from pancreatic, melanoma, colon, and breast cancer tissue (Table 1). However, compounds similar to **17 **had little effect on cell proliferation and were not pursued further in the current studies. Each of the compounds that inhibited cell proliferation showed interactions with ERK2 (Figure 2B) using fluorescence quenching assays that we previously described for the parent compound **76 **[17]. Inhibition of HeLa cell proliferation was dose dependent for compounds **76.2**, **76.3**, and **76.4 **(Table 2). Importantly, non-transformed epithelial cells that have been immortalized by stable expression of human telomerase reverse transcriptase (TERT) were less sensitive to the growth inhibitory effects of **76.3 **or **76.4 **(Table 2). TERT cells are useful cell culture controls for drug discovery as they retain relatively normal geno- and phenotypes and do not acquire cancer cell characteristics [34]. However, compounds **76 **and **76.2 **had similar potencies for inhibiting HeLa or TERT cell proliferation. Although the common chemical features of compounds **76.2**, **76.3**, **76.4 **and the parent compound **76 **include a thiazolidinedione core with an aminoethyl side group (Figure 1), the effects on cell proliferation suggest that the test compounds may have differences in their mechanisms of action.

**Table 1:** Effects of ERK targeted docking domain inhibitors on proliferation of cancer cell lines.

Compound	HeLa	SUM159	HCT-116	SKMEL-28	Panc-1
**76**	++	++	ND	++	++

**76.1**	-	++	-	-	ND

**76.2**	++	++	++	++	ND

**76.3**	++	++	++	++	ND

**76.4**	++	++	++	+	-

**76.5**	-	-	-	-	ND

**76.6**	-	-	-	-	ND

**76.7**	-	-	-	-	ND

**76.8**	-	-	-	-	ND

**76.9**	+	-	++	+	ND

**76.10**	-	-	-	-	ND

**17**	+	ND	ND	-	ND

**17.1**	-	-	-	-	ND

**17.2**	+	++	++	+	ND

**17.3**	-	-	-	-	ND

**17.4**	-	-	-	-	ND

**17.5**	-	-	-	-	ND

Transformed cell lines from cervical (HeLa), breast (SUM159), colon (HCT-116), skin (SKMEL-28), or pancreatic (Panc-1) tissue were treated for 7 days with 100 μM of the indicated test compound. The proliferation in treated cells is shown relative to control (DMSO only) following analysis using the colony formation assay. (-) ≤ 25%, (+) 25-75%, and (++) ≥75% inhibition; ND, No data

**Table 2:** Effects of ERK targeted docking domain inhibitors on cell proliferation.

	76		76.2		76.3		76.4	
	HeLa	TERT	HeLa	TERT	HeLa	TERT	HeLa	TERT
**25 μM**	20.1 ± 5.2^#^	21.7 ± 3.6^#^	73.7 ± 12.6	81.0 ± 8.1	58.2 ± 7.4^#^	112.9 ± 19.1	53.7 ± 6.0^#^	120.0 ± 26.5

**50 μM**	20.4 ± 4.1^#^	23.5 ± 12.0*	31.2 ± 12.9^#^	16.2 ± 4.0^#^	40.1 ± 8.0^#^	127.8 ± 12.8	36.3 ± 7.4^#^	48.1 ± 11.7^#^

**100 μM**	20.5 ± 5.9^#^	29.3 ± 12.1*	17.3 ± 7.1^#^	19.9 ± 12.5^#^	17.1 ± 6.1^#^	53.6 ± 6.8^#^	21.0 ± 6.3^#^	45.4 ± 12.6^#^

HeLa or non-transformed retinal pigment epithelial cells that stably express human telomerase reverse transcriptase (TERT) were seeded at 250 cells/mL and then treated for 7 days with 25-100 uM of the indicated test compound. The proliferation in treated cells is shown as a percentage of control cells (DMSO only set at 100%) following analysis using the WST-1 assay. Data represent mean ± SEM from 3 experiments. * and # indicates statistical significance compared to untreated controls, *p *≤ 0.05 and p ≤ 0.01, respectively

### Activation of intrinsic apoptosis by ERK inhibitors

**Figure 3:**
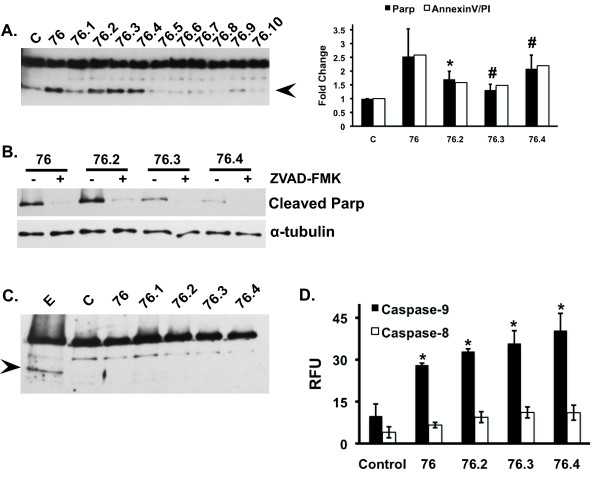
**Test compounds induce the intrinsic apoptosis pathway**. HeLa (**A**) or TERT (**C**) cells were treated in the absence or presence of test compounds (50 uM) for 5 hours (HeLa) or 16 hours (TERT). Cell lysates were immunoblotted for total and cleaved PARP (arrowhead). Graph on the right shows densitometry quantification of cleaved PARP to α-tubulin ratios and annexinV/PI staining relative control. PARP data represents the mean ± SEM from four independent experiments; annexinV/PI data are representative of two independent experiments. (**B**) HeLa cells were treated with 50 uM of the indicated compound for 5 hours and assayed for cleaved PARP in the presence and absence of the general caspase inhibitor ZVAD-FMK. (**D**) HeLa cells were treated in the absence or presence of 50 uM of the indicated compound for 5 hours and assayed for caspase 8 or 9 activity. Data represent the mean ± SEM from 3 independent experiments. * and # indicates statistical significance compared to untreated controls, *p *≤ 0.05 and *p *≤ 0.01, respectively. C, Control; E, Etoposide

To evaluate whether the changes in proliferation were linked to an apoptotic response, cells were treated with test compounds at 50 μM, which resulted in at least 50% inhibition of HeLa cell proliferation (Table 2), and examined for cleavage of poly-ADP ribose polymerase (PARP) as a marker of apoptosis. Compounds **76**, **76.2**, **76.3**, and **76.4 **induced PARP cleavage and annexin V/PI staining after 5 hours exposure (Figure 3A) and this response could be blocked with a general caspase inhibitor, Z-VAD-FMK (Figure 3B). In contrast, PARP cleavage was not observed in TERT cells even after longer (16 hour) exposures to the test compounds (Figure 3C). To determine whether the test compounds induced apoptosis through extrinsic or intrinsic mechanisms, caspase-8 and 9 activities were examined. Compounds that induced PARP cleavage also induced caspase-9 activity but had little effect on caspase-8 activity indicating that ERK-targeted test compounds activate an intrinsic apoptosis pathway (Figure 3D).

### Test compounds inhibit ERK-mediated phosphorylation of p90 Rsk-1 and Bad proteins

**Figure 4:**
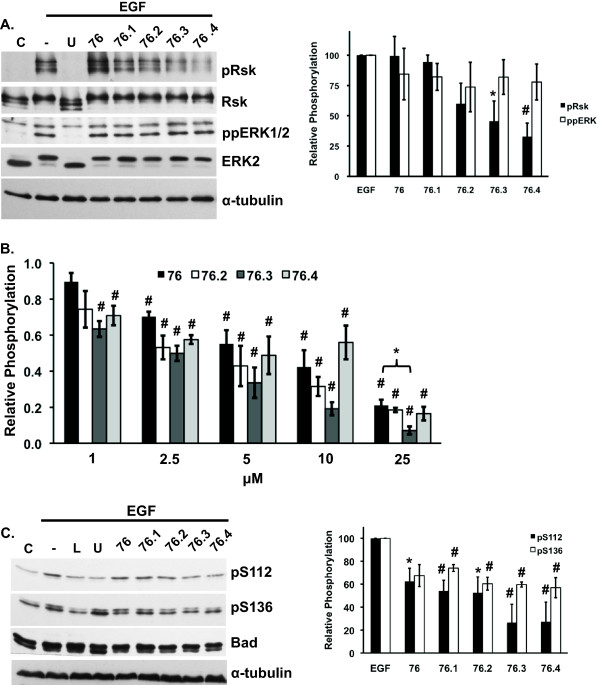
**Test compounds inhibit ERK-mediated phosphorylation of p90Rsk-1 and Bad**. (**A**) HeLa cells were pre-treated for one hour in the presence or absence of 50 uM of the indicated test compounds and then stimulated with EGF (25 ng/ml) for 10 minutes. Immunoblots of phosphorylated p90Rsk-1 (pRsk), total Rsk (Rsk), phosphorylated ERK1/2 (ppERK), and total ERK2 (ERK2). α-tubulin was used as a loading control. Graph shows densitometry quantification of pRsk-1 to total Rsk or ppERK2 to ERK2 ratios. Data represents the mean ± SEM from three independent experiments. (**B**) *In vitro *kinase assays examining ^32^P incorporation into p90Rsk-1 following incubation with active ERK2 and γ-^32^P-ATP for 60 min. in the absence or presence of 1-25 μM of test compounds. Relative phosphate incorporation was quantified by phosphoimager analysis. (**C**) HeLa cells were serum starved overnight and pre-treated for 1 hr with 50 uM indicated test compounds, 10 mM U0126 (U), or 25 mM LY294002 (L) prior to stimulation with or without EGF (25 ng/ml). Immunoblot analysis of Bad phosphorylated on Ser112 (pS112) or Ser136 (pS136) and total Bad. α-tubulin was used as a loading control. Data represents the mean ± SEM from three independent experiments. * and # indicates statistical significance compared to EGF-only treatment (A and C) or untreated controls (B), *p *≤ 0.05 and *p *≤ 0.01, respectively. C, untreated control; (-) EGF treated control.

ERK proteins provide a survival advantage by directly or indirectly phosphorylating and inactivating the Bcl-2 family members of pro-apoptotic proteins [3,4]. Bad is a pro-apoptotic Bcl-2 family member that is inactivated by phosphorylation at S112 by p90RSK-1 and S136 by Akt [4,35]. The intrinsic mechanisms of apoptosis induction by the test compounds were first examined by evaluating ERK-mediated phosphorylation of p90Rsk-1. Phosphorylation of p90Rsk-1 was inhibited by 40, 54, and 67% in the presence of 50 μM of test compound **76.2**, **76.3**, and **76.4**, respectively (Figure 4A). As expected, **76.1**, which was less effective in inhibiting cell proliferation, did not inhibit p90Rsk-1 phosphorylation (Figure 4A). It was also somewhat surprising that **76 **did not affect p90Rsk-1 phosphorylation at this dose although 2 fold higher doses did inhibit p90Rsk-1 phosphorylation by 50% in our previous studies [17]. None of the compounds caused a statistically significant inhibition of ERK1/2 activation. These compounds are predicted to target ERK2 near the CD domain and may affect ERK activation by upstream MEK1/2 proteins, which reportedly interact with ERK through the CD domain [36]. Nonetheless, compounds **76.3**, and **76.4 **appeared to show selectivity for inhibiting ERK-mediated phosphorylation of p90Rsk-1 without inhibiting ERK activation. Similarly, **76.3 **was the most potent inhibitor of phosphate incorporation into p90Rsk-1 as measured using *in vitro *kinase assays and statistically more potent than the parent compound **76 **at 25 μM (Figure 4B).

We next examined Bad phosphorylation at S112 and S136 in HeLa cells treated with test compounds. Compounds **76.3 **and **76.4 **appeared the most potent inhibitors and reduced EGF-induced S112 phosphorylation by ~75-80% (Figure 4C). In contrast, S136 phosphorylation in the presence of these compounds was inhibited by ~40%, which supports a selective inhibition of the ERK/p90Rsk-1 over Akt signaling. As controls, the MEK1/2 inhibitor, U0126, inhibited phosphorylation of S112 but not S136, whereas the PI3K inhibitor, LY294002, inhibited phosphorylation of both S112 and S136 (Figure 4C) consistent with studies suggesting that phosphorylation of S136 induces a conformational change in Bad to promotes access to kinases that phosphorylate other sites [37].

### Test compounds affect the expression of pro- and anti-apoptotic proteins Bad and Mcl-1

**Figure 5:**
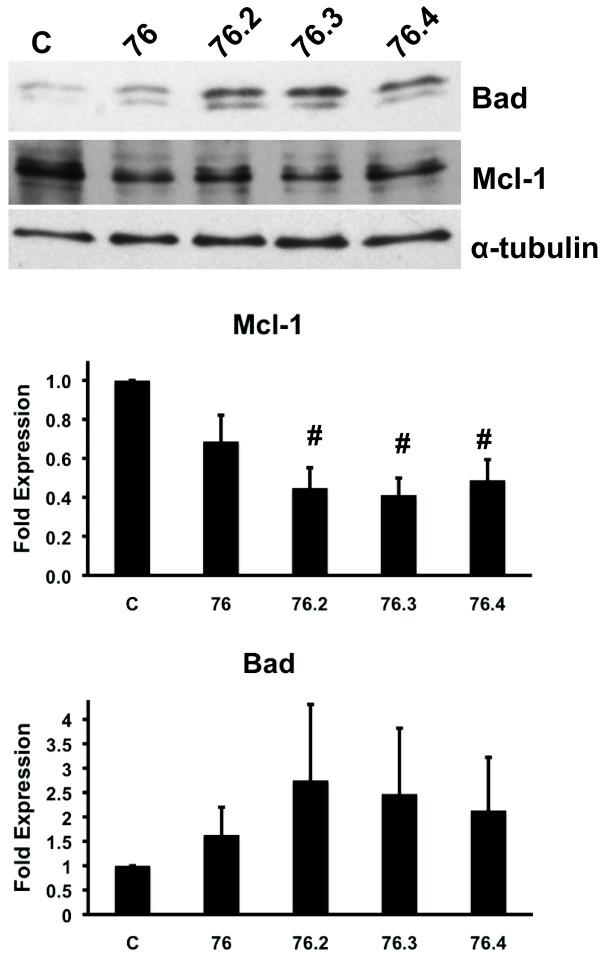
**Test compounds affect the expression of the pro- and anti-apoptotic proteins, Bad and Mcl-1, respectively**. Total Bad and Mcl-1 immunoblots from lysates collected from HeLa cells treated for 16 hour with 50 μM of indicated test compounds (top panel) and fold expression quantified by densitometry (bottom panel). α-tubulin was used as a loading control. Data represent the mean ± SEM of three independent experiments. # indicates statistical significance compared to untreated controls, *p *≤ 0.01. C, Control

We also examined the test compounds effects on protein expression of Bad as well as Mcl-1, which is stabilized following ERK phosphorylation [38]. Although a two-fold increase in total Bad expression was observed in cells treated for 16 hours with the active test compounds (Figure 5), these data were not statistically significant. Nonetheless, these findings support previous studies showing increased Bad expression following inhibition of the ERK pathway with the MEK1/2 inhibitor, PD98059 [39]. However, the test compounds caused a statistically significant decrease in the expression of the anti-apoptotic protein Mcl-1 (Figure 5). Thus, these data indicate that the test compounds may sensitize cells to undergo apoptosis by increasing the expression of pro-apoptotic proteins and decreasing the expression of anti-apoptotic proteins, which are regulated by the ERK pathway.

### Test compounds inhibit caspase-9 phosphorylation

**Figure 6:**
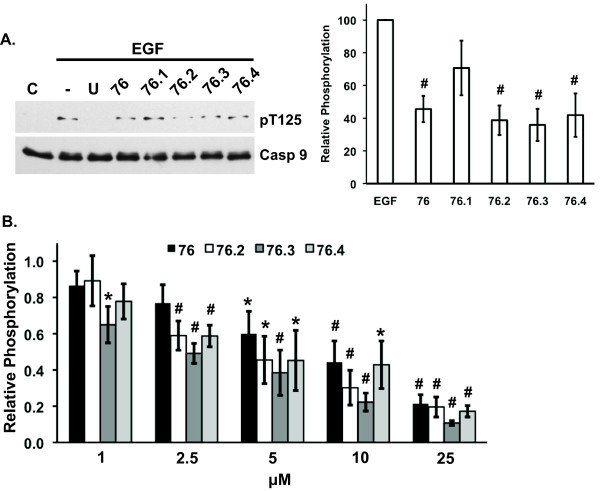
**Test compounds inhibit ERK-mediated phosphorylation of caspase-9 in cells and in *in vitro *kinase assays**. (**A**) HeLa cells transfected with caspase-9 (C287A) were pre-treated for 1 hour with 50 uM of indicated test compound, stimulated with EGF (25 ng/ml), and immunoblotted for phosphorylated caspase-9 (pT125) or total caspase-9. Graph shows the mean ± SEM for relative phosphorylation as determined by densitometry scanning from 3 independent experiments. (**B**) *In vitro *kinase assays examining ^32^P incorporation into caspase-9 following incubation with active ERK2 and γ-^32^P-ATP for 60 min. in the absence or presence of 1-25 μM of the indicated test compound. Relative phosphate incorporation was quantified by phosphoimager analysis. Data show the mean ± SEM of three independent experiments. * and # indicates statistical significance compared to EGF-only treatment (**A**) or untreated controls (**B**), *p *≤ 0.05 and *p *≤ 0.01, respectively. C, untreated control; (-), EGF treated control; U, U0126

ERK phosphorylation of caspase-9 inhibits caspase-9 proteolytic activity and provides another mechanism by which activated ERK proteins mediate cell survival [2]. The effects of test compounds on ERK-mediated phosphorylation of caspase-9 on T125 were examined by immunoblotting lysates from cells where the ERK pathway was stimulated with EGF. Apoptosis-inducing test compounds **76**, **76.2**, **76.3**, and **76.4**, inhibited caspase-9 phosphorylation by 50-60% in treated cells (Figure 6A). Next, active ERK2 was incubated with caspase-9 protein in the absence or presence of test compounds and phosphorylation was examined using *in vitro *kinase assays. Figure 6B shows that compounds **76**, **76.2**, **76.3**, and **76.4 **inhibited ERK-mediated phosphorylation of caspase-9 in a dose dependent manner. While **76.3 **appeared to be the more potent inhibitor of caspase-9 phosphorylation, there was no statistical difference between compounds at any of the doses.

## Discussion

The goal of these studies was to characterize a class of novel ERK inhibitor compounds being developed in our laboratory that inhibit cell proliferation. Our findings demonstrate the induction of an apoptotic response by structurally-related compounds that inhibit ERK regulation of pro-apoptotic proteins. Aberrant activation of ERK signaling has been well-documented and provides a survival advantage in a number of cancers by inactivating pro-apoptotic proteins [40]. Thus, selective inhibition of ERK anti-apoptotic functions is a potential approach to sensitize cancer cells to chemotherapeutic agents. One important feature of the active test compounds is that some appear to be more selective for decreasing cell proliferation and inducing apoptosis in transformed cells as compared to non-transformed cells (Table 2 and Figure 3). This finding is in agreement with our previous studies indicating that the test compounds did not affect the life span or cause general toxicity in a *C. elegans *whole organism model [32]. Interestingly, we also have not observed overt toxicity when treating mice harboring B-cell lymphomas with compound **76 **[41]. Coincidentally, these *in vivo *studies also demonstrated that B-cell lymphoma tumor burden was significantly reduced by **76**. However, we predict that the significance of the ERK-targeted inhibitors in reducing tumor burden will best be realized using potentially more potent compounds, such as **76.3**, in combination with other chemotherapeutic agents.

Our data support a mechanism by which the ERK-targeted compounds prevent inactivation of caspase-9 and p90Rsk-1 phosphorylation of Bad (Figures 4 and 6). Both caspase-9 and p90Rsk-1 contain D-domains that are thought to form contacts with the CD domain of ERK proteins [1,24]; the region predicted to be targeted by the test compounds. Structural studies underway are determining the site of interactions between the compounds and ERK proteins to better understand the mechanisms of inhibition and identify chemical features that can be modified to improve inhibitor binding and efficacy. A region analogous to the CD domain of ERK can be found on other MAP kinases family members including p38 and JNK [29,42]. The subtle differences between these regions are determinants of substrate selectivity between the MAP kinases. In addition, differences in the residues associated with the D-domains of MAP kinase interacting proteins can confer selectivity. For example, the differences in D-domains on MAP kinase kinase (MKK) proteins appear to be important for determining what MAP kinase the MKK isoform will target [43].

The p90Rsk-1 protein is a major regulator of ERK-mediated cell survival [5] and, like Bad, other p90Rsk-1 substrates may be affected by the test compounds. For example, inhibition of p90Rsk-1 could also restore activity of the death associated protein kinase (DAPK), which is a pro-apoptotic tumor suppressor protein that is phosphorylated and inactivated by p90Rsk-1 [44]. Moreover, it is recognized that other survival proteins may be affected by the test compounds and involved in mediating the apoptotic response observed. While some inhibition of PI3K/Akt signaling may have occurred with the test compounds (data not shown), these effects were not to the extent of inhibition of Akt substrates with the PI3K inhibitor, LY294002. In addition, the activation of other growth related proteins like ERK5 and EGF receptor (data not shown) were not affected, which further supports the mechanism of apoptosis induction by the test compounds involves inhibition of ERK1/2 signaling.

Another objective of this study was to determine whether improvements in potency and selectivity of the parent compound (**76**) could be achieved by examining structurally similar compounds. Cell based and *in vitro *kinase assays in figures 4 and 6 suggest that compound **76.3 **has higher potency than **76**. Thus, these studies have identified a class of structurally similar compounds that have similar biological effects and chemical features that can be modified to improve potency. Studies are now aimed at performing a more detailed structure activity relationship to identify the mode of the compound's interaction with ERK, improve potency, and determine whether other substrate proteins are affected. The present results also indicate the potential utility of using LMW compounds that inhibit protein-protein interactions in a therapeutic setting. While it has traditionally been assumed that small compounds would not be effective inhibitors of protein-protein interactions [45,46], recent studies involving the transcription factor BCL6 indicate that protein-protein interactions can be targeted *in vivo *with LMW compounds [47]. The efficacy of the present compounds as inhibitors of ERK-substrate interactions and their activity in a xenograft mouse model [41] further indicate that protein-protein interactions are valid therapeutic targets of LMW compounds.

The specific targeting of the ERK1/2 pathway to treat cancer has been best studied using MEK1/2 inhibitors (reviewed in [48]). Although a number of preclinical studies showed very good efficacy of MEK inhibitors for reducing cancer cell proliferation and inducing apoptosis, patient responses in clinical trials have been minimal with these compounds as single agents. Thus, MEK inhibitors in combination with other chemotherapeutic drugs will likely be more effective. A potential problem with targeting MEK for cancer therapy is the development of drug resistance, possibly through compensatory mechanisms involving elevated expression and activity of MEK/ERK proteins [49]. Thus, the approach to completely inhibit MEK/ERK signaling may be counterproductive in the long term. In contrast, the use of compounds that selectively inhibit ERK functions involved in cell survival may have the potential to sensitize cancer cells to other chemotherapeutic agents without inducing the compensatory pathways that lead to acquired drug resistance.

## Conclusion

We have identified a class of LMW compounds that are selective for ERK docking domains involved in regulating interactions with substrates that promote cell proliferation and survival. These new compounds inhibit transformed cell proliferation and induce apoptosis, in part, by inhibiting substrate phosphorylation events that regulate pro- and anti-apoptotic proteins. Future optimization of the chemical features of the new lead compounds may provide more potent and selective molecules that can improve the efficacy of existing chemotherapeutic agents by disabling some of ERK1/2 protein functions.

## Competing interests

The authors declare that they have no competing interests.

## Authors' contributions

SB participated in study design, performed the experiments and statistical analysis as well as writing the manuscript. RD performed the colony formation assays. UP and AM designed the ERK targeted docking domain inhibitors. SS provided valuable insight and critically reviewed the manuscript. AM and PS participated in the conception and study design. PS coordinated the study and the writing of the manuscript. All authors approved the final manuscript.

## Pre-publication history

The pre-publication history for this paper can be accessed here:

http://www.biomedcentral.com/1471-2407/11/7/prepub
